# First in vitro cell co-culture experiments using laser-induced high-energy electron FLASH irradiation for the development of anti-cancer therapeutic strategies

**DOI:** 10.1038/s41598-024-65137-7

**Published:** 2024-06-27

**Authors:** Stefana Orobeti, Livia Elena Sima, Ioana Porosnicu, Constantin Diplasu, Georgiana Giubega, Gabriel Cojocaru, Razvan Ungureanu, Cosmin Dobrea, Mihai Serbanescu, Alexandru Mihalcea, Elena Stancu, Cristina Elena Staicu, Florin Jipa, Alexandra Bran, Emanuel Axente, Simion Sandel, Marian Zamfirescu, Ion Tiseanu, Felix Sima

**Affiliations:** 1 National Institute for Laser, Plasma and Radiation Physics (INFLPR), 409 Atomistilor Street, RO-077125 Magurele, Romania; 2 Department of Molecular Cell Biology, Institute of Biochemistry of the Romanian Academy, 296 Splaiul Independentei, 060031 Bucharest, Romania

**Keywords:** Laser-produced plasmas, Radiotherapy, Wide-field fluorescence microscopy

## Abstract

Radiation delivery at ultrahigh dose rates (UHDRs) has potential for use as a new anticancer therapeutic strategy. The FLASH effect induced by UHDR irradiation has been shown to maintain antitumour efficacy with a reduction in normal tissue toxicity; however, the FLASH effect has been difficult to demonstrate in vitro. The objective to demonstrate the FLASH effect in vitro is challenging, aiming to reveal a differential response between cancer and normal cells to further identify cell molecular mechanisms. New high-intensity petawatt laser-driven accelerators can deliver very high-energy electrons (VHEEs) at dose rates as high as 10^13^ Gy/s in very short pulses (10^–13^ s). Here, we present the first in vitro experiments carried out on cancer cells and normal non-transformed cells concurrently exposed to laser-plasma accelerated (LPA) electrons. Specifically, melanoma cancer cells and normal melanocyte co-cultures grown on chamber slides were simultaneously irradiated with LPA electrons. A non-uniform dose distribution on the cell cultures was revealed by Gafchromic films placed behind the chamber slide supporting the cells. In parallel experiments, cell co-cultures were exposed to pulsed X-ray irradiation, which served as positive controls for radiation-induced nuclear DNA double-strand breaks. By measuring the impact on discrete areas of the cell monolayers, the greatest proportion of the damaged DNA-containing nuclei was attained by the LPA electrons at a cumulative dose one order of magnitude lower than the dose obtained by pulsed X-ray irradiation. Interestingly, in certain discrete areas, we observed that LPA electron exposure had a different effect on the DNA damage in healthy normal human epidermal melanocyte (NHEM) cells than in A375 melanoma cells; here, the normal cells were less affected by the LPA exposure than cancer cells. This result is the first in vitro demonstration of a differential response of tumour and normal cells exposed to FLASH irradiation and may contribute to the development of new cell culture strategies to explore fundamental understanding of FLASH-induced cell effect.

## Introduction

New radiotherapeutic treatment schemes for solid cancers have been developed and tested to provide a differential response of targeted tumour cells compared with that of normal cells. Radiotherapy delivered via ultrahigh dose rate (UHDR) irradiation, known as FLASH, has demonstrated similar efficacy in affecting tumour progression to conventional dose rate radiotherapy while reducing the toxicity of the healthy tissue encountered in standard treatment^1,2^. FLASH irradiation is defined by dose rates greater than 40 Gy/s; this is a few orders of magnitude greater than those of conventional treatments carried out at doses of approximately 0.03 Gy/s. The FLASH-induced effect was validated in preclinical experiments with electron^3^ and proton beams^4^. However, the biological mechanisms underlying the FLASH effect are poorly understood. FLASH irradiation may cause the consumption of oxygen around the tumour and produce temporary local hypoxia, reducing the development of fast-proliferating cancer cells^5^. Other studies proposed that FLASH irradiation may be involved in the reduction of hydrogen peroxidase, which is correlated with the modification of reactive oxygen species (ROS) production in tissues and affects the biological response^6^. In the last decade, several laboratories have modified linear accelerators to generate UHDRs and studied the FLASH effect^1,7^. To date, the UHDR-induced FLASH effect has been demonstrated in several in vivo cancer animal models.

Compared with photon-based therapy, the use of very high-energy electrons (VHEEs) with energies greater than 50 MeV is an alternative therapeutic solution for deep-seated tumours due to the potential improvement in the dose distribution. This potential improvement has been intensively explored in recent years and new, more effective radiotherapeutic strategies were developed^8–10^.

High-intensity laser-driven acceleration of VHEE represents a new approach that may be an alternative solution for radiotherapeutic treatments with electrons. Indeed, VHEEs with dose rates as high as 10^13^ Gy/s^11–14^ are achievable by laser-plasma accelerators (LPAs) with quasi-monoenergetic beams^15^. This ultrafast radiation biology may be considered a new emerging interdisciplinary field, in combination with advances in ultrashort radiation sources, high-energy chemistry, and anticancer therapy^16^. The unique characteristics of LPA sources rely on its wider accessibility with respect to conventional accelerators, and LPA sources have practical advantages that could have a major impact on the development of new radiotherapeutic sources. Notably, an LPA system capable of producing electrons with energies of hundreds of MeV exhibits a footprint of tens of square metres, which is much less than that of a similar conventional linear accelerator (LINAC). In addition, a few hundred centimetres of an electron pathway demonstrates the system's ability to provide a table-top compact setup, which is very useful for radioprotection procedures. These electron beams were found to be suitable for delivering a high dose peak on the propagation axis with deep penetration combined with a sharp and narrow transverse penumbra^17^. Initial studies with LPA were devoted to characterizing dosimetric aspects^18–22^.

LPA’s has been proposed as a new platform for VHEE-induced FLASH irradiation^15^, but very few experiments have explored the feasibility of this technique^23^. The first irradiation experiments for in vivo studies involved more than 50 mice, using a dose rate exceeding 1 Gy/min at electron energies above 5 MeV; this energy is considered stable and provides reliable operation of the system^24^. In addition, only a few other studies have attempted to assess the effects of LPA VHEE on cell damage. LPA VHEE was shown to cause DNA damage in skin carcinoma cells exposed to a very high dose rate from a single, ultrashort bunch of high-energy electrons^16^. Additional experiments with such short durations of LPA beams may help discover novel phenomena in radiobiology, particularly at the early phase of the damage induced to cell structure by ionizing radiation.

Here, we present the first in vitro experiments carried out with cell co-cultures exposed to LPA electrons generated by the interaction of a high-intensity laser beam with gas jets. Melanoma cancer cells and normal melanocyte co-cultures were irradiated with either VHEE produced by LPA or pulsed X-ray beams. The potential differential responses of cancer cells and normal cells to the proposed irradiation setup available in our facility were evaluated by measuring induced double-strand DNA breaks in comparison to X-rays. First step necessary to determine if a certain radiation source and applied protocol has the potential to become one of the elements in the oncologists’ toolbox to be used in the fight against tumours is to evaluate its DNA damage inducing capacity^25^. This step was crucial for demonstrating the quality potential of VHEEs generated by LPA, thus faciliting new opportunities for extensive utilization in clinical studies.

## Results

**Figure 1:**
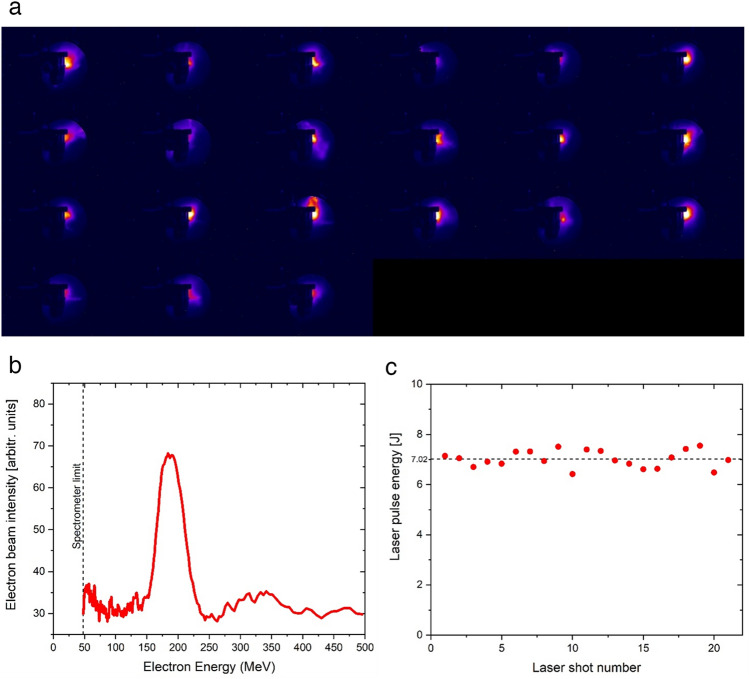
(**a**) Raw images of the scintillating screen together with the cell containers exposed to laser-plasma accelerated electrons; (**b**) Energy spectrum of electrons; (**c**) Laser energy stability during the 21 shots session.

Here, we present the results obtained in vitro in two irradiation sessions in which the conditions were similar, and these sessions are further referred to as experiments PW1 and PW2. The electron spectrum was quasi-monoenergetic with a peak energy E_peak_ of ~ 190 ± 40 MeV (Fig. 1b). Values under 50 MeV are missing due to the constructive spectrometer limit. The number of applied laser shots varied from one experiment to another, and a fixed total dose of 150 mGy was maintained. This dose was measured using an ionization chamber located 5 cm behind the stack of containers and thermoluminescent dosimeters (TLDs) (as depicted in Fig. 4). The array of images presented in Fig. 1a shows the LANEX images acquired during a session in which 21 shots were applied. The laser energy was highly stable over the 21 shots, with an E_laser_ (mean ± s.d.) = 7.02 ± 0.34 J (see Fig. 1c and movie in the Supplementary Information).

The doses measured by the three independent dosimetry systems for each of the two (I, II) irradiation sessions were as follows: (i) I. 149.9 mGy and II. 154.8 mGy by the ionization chamber; (ii) I. 1107.28 mGy, 627.48 mGy, and 400.97 mGy; II: 1263.08 mGy, 748.28 mGy, and 429.65 mGy by the three TLDs placed in front, middle, and behind the cell containers, respectively; and (iii) I. 195 mGy and II. 170 mGy by Gafchromic films (GFs) placed behind the stack. The differences in the measured dose were potentially the result of the broad energy spectrum of the electron beam accelerated by high-power laser target interactions. The highest values recorded by the TLD readings (highest low limit sensitivity) potentially indicate the presence of low-energy electrons transient through and/or stopped within the samples and not registered by the Gafchromic films and Advance Markus ionization chamber. A discussion on this aspect is presented in the SI (Supplementary Information). Additionally, the three dosimetry systems were placed at different depths along the electron beam propagation axis and involved different radiation-sensitive media, which accounts for the variations in dose.

**Figure 2:**
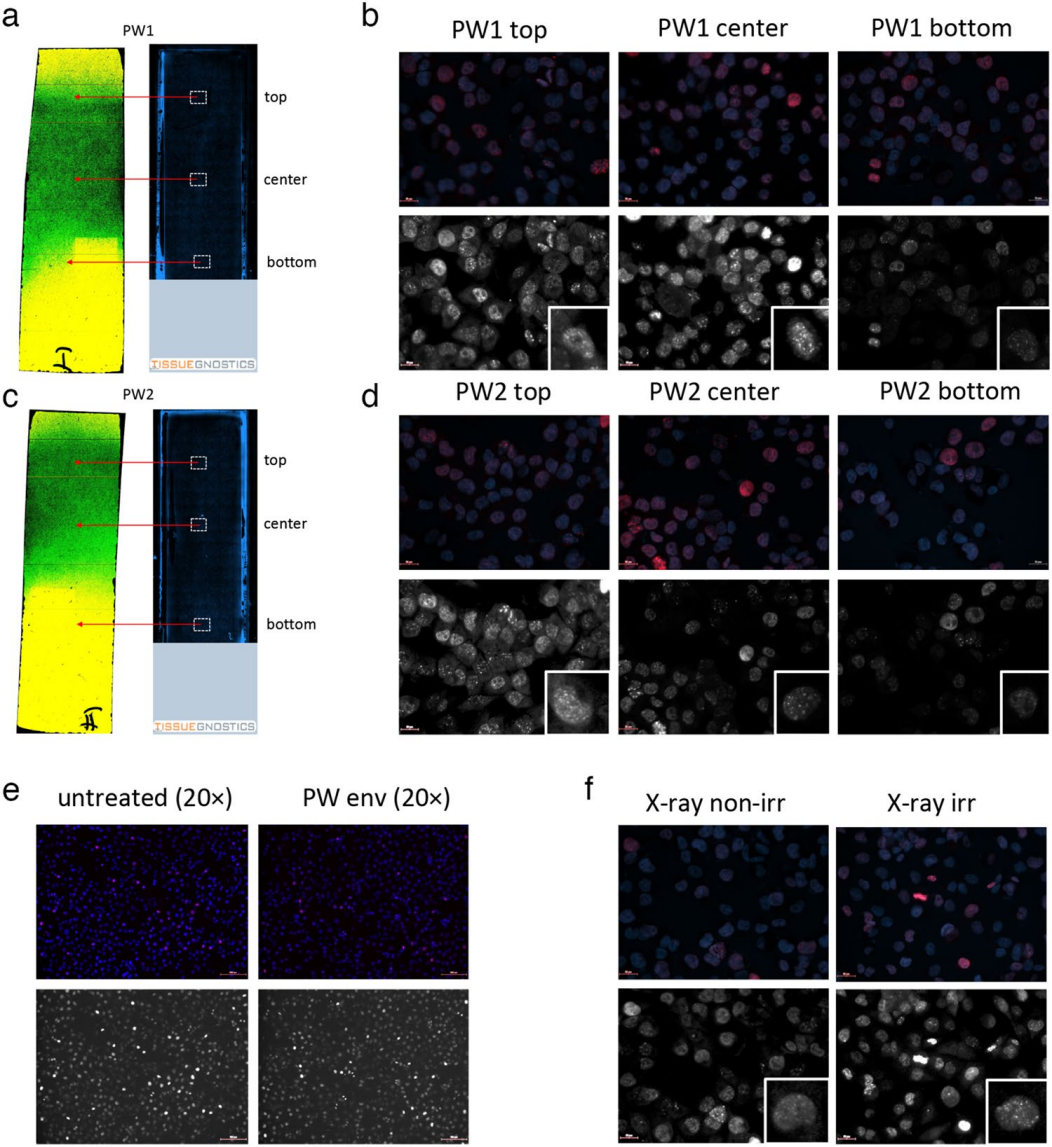
Analysis of the A375 melanoma and NHEM co-cultures upon laser-plasma accelerated electron exposure during 2 sequentially performed irradiation experiments (PW1, PW2) versus pulsed X-ray irradiation. (**a**,**c**) Correspondence between the irradiated GF fingerprint and selected areas of the SlideFlasks for image cytometry analysis (5 × magnification preview mode). (**b**,**d**,**e**,**f**) DNA damage response revealed as p-γ-H2AX foci at 30 min post-exposure to PW or X-ray-generated radiation versus non-exposed (untreated) or mock-exposed (PW env) negative controls. Immunofluorescence microscopy images of overlapping DAPI and TxRed signals (top), as well as grayscale images of the TxRed signal (bottom) of a representative field of view (FOV) under each irradiation condition, are shown. The p-γ-H2AX foci are visible in the detailed insets (**b**,**d**,**f**—bottom right). Scale bar = 20 μm (**b**,**d**,**f**) or 100 μm (**e**).

**Figure 3:**
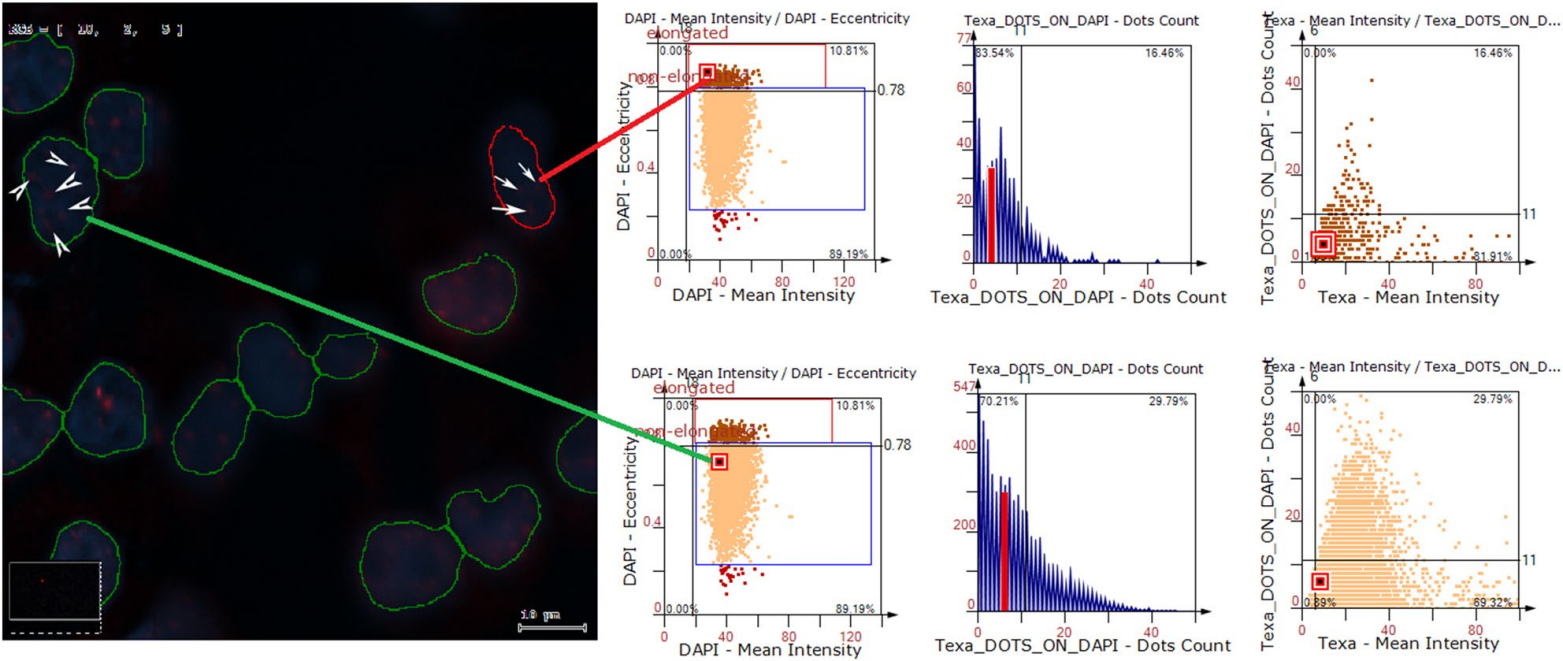
Image cytometry analysis strategy of irradiated A375:NHEM co-cultures. Upon labelling with antibodies against p-γ-H2AX (red) and with Hoechst for nuclear DNA (blue), samples were automatically scanned using the TissueFAXSiPlus imaging system. After regions of interest (ROIs) reconstitution out of stitched individual fields of view (FOV), the obtained images were imported into the TissueQuest image quantification software. Each cell was identified based on nuclear DAPI signal by the segmentation algorithm. Next, scattergrams were generated based on DAPI-Eccentricity versus DAPI-Mean Intensity profiles and cell lines differentiated based on nuclear elongation (eccentricity): elongated nuclei of NHEM were gated (red gate) based on higher eccentricity values, while non-elongated A375 nuclei were gated (blue gate) based on low eccentricity values. Using forward gating, one can identify the scattergram displayed signals for each cell in a population. Here, we exemplify a NHEM nucleus encircled in red and an A375 nucleus encircled in green and their corresponding signals in the scattergrams (high vs. low elongation). In order to quantify p-γ-H2AX mean fluorescence intensity (MFI) and number of foci per nucleus, TxRed signals from the nuclear mask were quantified using 2 parameters: Texa-Mean Intensity and Texa_DOTS_ON_DAPI-Dots Count, respectively, visible on the axes. Histograms displaying frequency distribution of number of foci per nucleus are displayed in blue for each cell population. Also, scattergrams of number of foci versus p-γ-H2AX expression level can be generated. Here, red bars and dots identify the selected nuclei in the picture. Foci are indicated by white arrows (in NHEM) or arrowheads (in A375).

**Figure 4:**
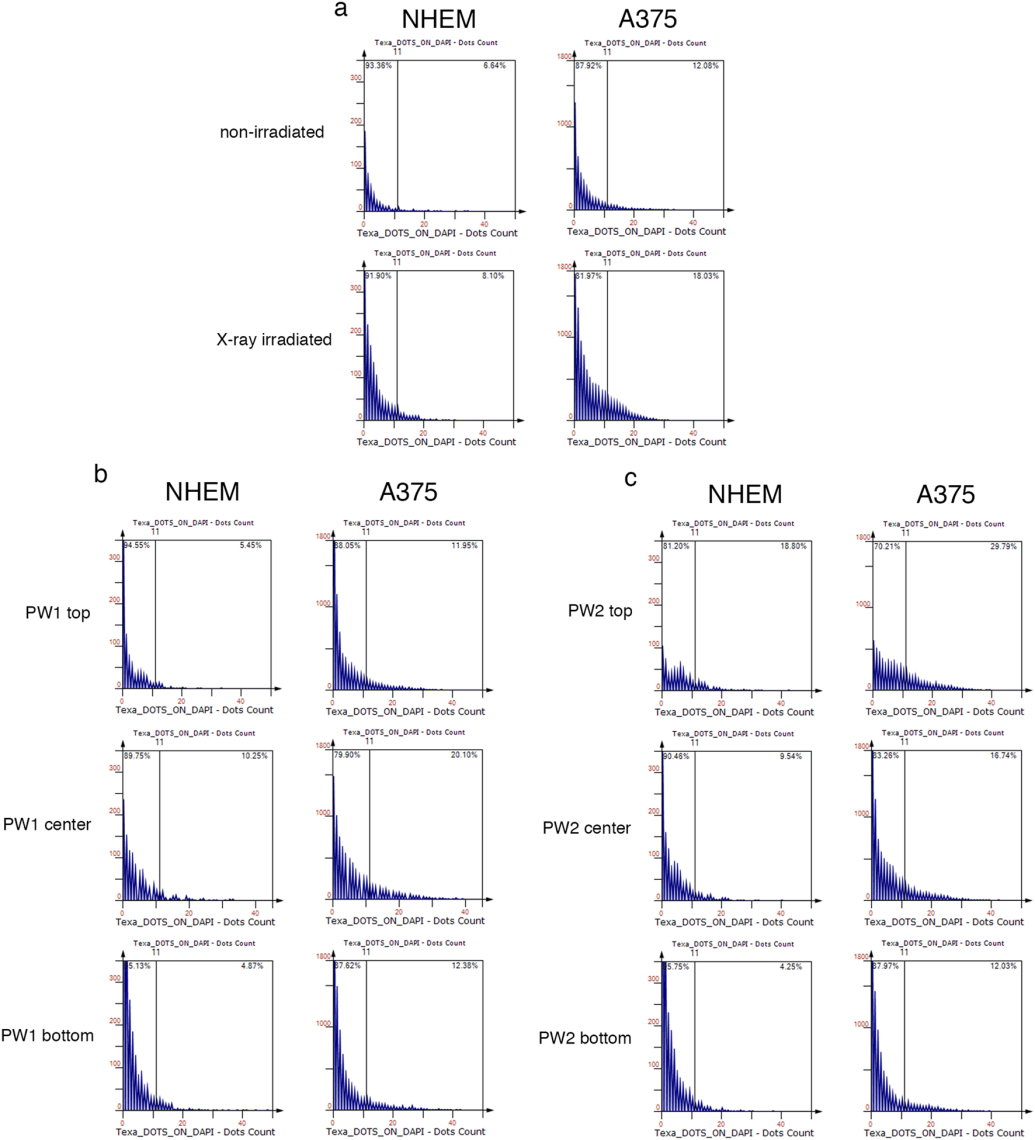
Frequency distribution of number of foci per nucleus for cells in each region of interest (ROI) analysed of the X-ray (**a**), PW1 (**b**), and PW2 (c) irradiated samples. Cells with 0 up to 44 foci per nucleus were identified in the irradiated and non-irradiated samples (Texa_DOTS_ON_DAPI – Dots count parameter). A threshold is depicted as baseline detected in normal melanocytes (NHEM) in the absence of radiation exposure (a—top left). One can observe an increase in the fraction of cells presenting higher number of p-γ-H2AX foci per nucleus in samples exposed to different irradiation conditions (**a**–**c**), as compared to non-irradiated control (a – top histograms) (n = 4473–10,629 analysed nuclei).

**Figure 5:**
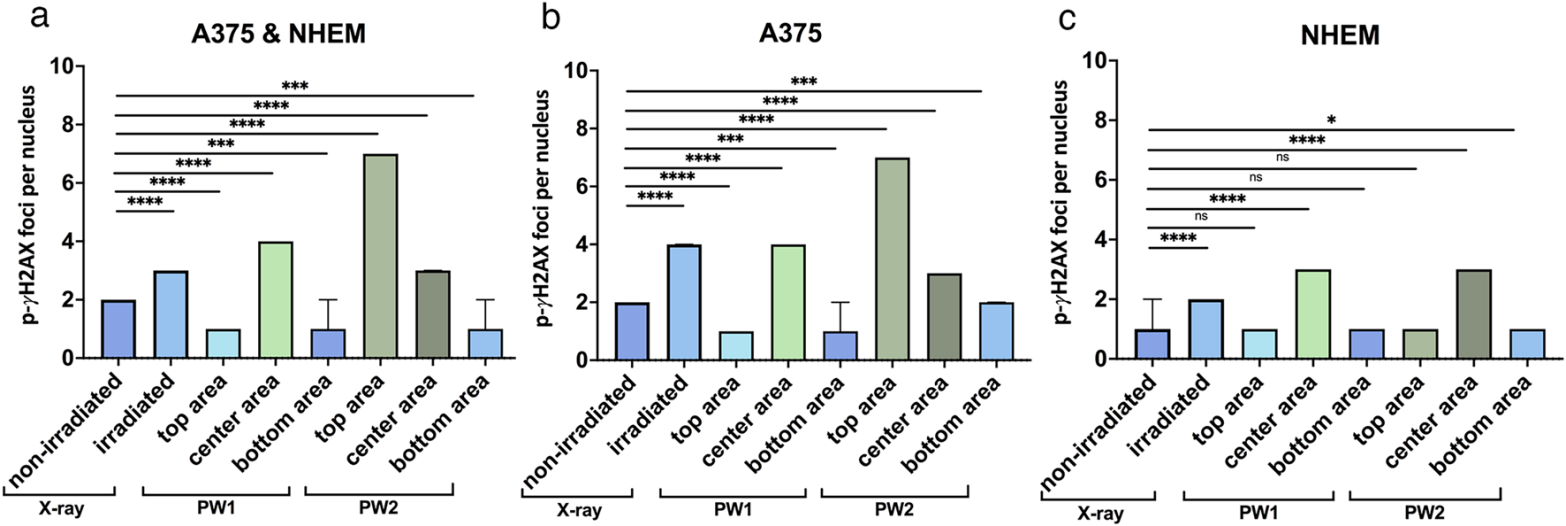
Comparison of “number of p-γ-H2AX foci per nucleus” values for each tested irradiation condition. The bar graphs show the comparisons between treatments for the overall A375 and NHEM co-cultures (**a**), for the A375 cells in co-cultures (**b**), and for the NHEM cells in co-cultures (**c**). Sampled areas of PW-irradiated specimens and the standard pulsed X-ray-irradiated cells were compared to non-irradiated controls. Medians of each data set and 95% confidence intervals are displayed. The statistical differences were determined using the nonparametric Mann–Whitney test, (n = 4473–10,629 analysed nuclei, *p < 0.05, ***p < 0.001, ****p < 0.0001, *ns* = not significant).

We measured the immediate response of the radiation-resistant A375 melanoma cells and normal human epidermal melanocytes (NHEMs) 30 min after the exposure of cocultures to irradiation treatments, when p-γ-H2AX was shown to have its peak nuclear expression at DNA double-strand break (DNA-DSB) sites^26^. For this, two SlideFlasks containing NHEM: A375 co-cultures (seeded one day prior to the experiments in a 1:2 numeric ratio) were irradiated serially at an ultrahigh dose rate (UHDR) within the same session using the PW laser facility, while a third identical SlideFlask was irradiated using an optimized pulsed X-ray setup with a conventional dedicated device, and a forth remained non-irradiated. GFs were attached to the bottom of each slide to record the dose and pattern of exposure of each replicate. After 30 min of incubation at 37 °C, the cells were fixed for immunofluorescence analysis of the induced DNA damage foci. In parallel, a sample was treated for 24 h with 2 μM cisplatin (CisPt) to serve as a positive control for foci induction (Figure SI 3, A–C). DNA DSBs are produced in response to stress-induced either by chemical (*e.g*., chemotherapeutics, such as CisPt) or radiological (*e.g.,* X-ray exposure) treatments. If not resolved by cell molecular repair mechanisms, the affected cells are stalled from proliferation and later die by the induction of apoptosis or alternative death pathways^27^. Hence, the occurrence of DNA damage foci is the first consequence of effective stress inflicted upon the targeted cells. We sampled 3 areas from the top, centre, and bottom of the vertically positioned slide to analyse the effect produced by PW laser-induced electron irradiation (Fig. 2 a and c; the developed GF *vs*. TissueFAXS scanned preview). Upon scanning the ROIs with a high magnification (63 ×) objective, the number of p-γ-H2AX foci in each nucleus was quantified using an image cytometry approach (Fig. 3). Notably, the DNA damage effect observed by foci quantification in each of the 3 selected regions of each PW irradiation exposed SlideFlask (Figs. 4 and 5) was consistent with the gradient pattern detected by GF dose mapping (Fig. 2a and c; the developed GF *vs.* TissueFAXS scanned preview).

The first irradiation procedure (sample PW1) induced the greatest effects in the centre of the slide (Fig. 2b, Fig. 4b and Fig. 5a), while the second (sample PW2) procedure impacted mostly the top of the irradiated sample (Fig. 2d, Fig. 4c and Fig. 5a). Consequently, these areas had an increased frequency of nuclei with high foci counts compared to the other two regions in the corresponding samples (Fig. 4 and Fig. 5a). The top area of the PW2 slide had a greater tendency for DNA-damaged nuclei than the other investigated areas; the centre of the PW1 slide showed the most comparable effect to that of X-ray irradiation (Fig. 2b *vs.* f., Fig. 4b *vs.* a, and Fig. 5a, cell co-culture), and this was considered to be a positive control for radiation-induced DNA-DSB. Interestingly, upon analysing each cell type individually (Fig. 5b and c, respectively), we observed that PW2 exposure had a different pattern of impact on NHEMs than on the A375 melanoma cells; here, the normal cells were affected more in the centre area of the slide than in the top area, when compared to cancer cells. This was observed only for this irradiation condition. Hence, NHEM cells had increased DNA damage in the centre of the slide, irrespective of the PW1 and PW2 irradiation patterns. The environment in the PW room did not induce supplementary DNA damage compared to the baseline observed in untreated control cells (Fig. 2e).

## Discussion

Approximately 50% of all cancer patients undergo a radiotherapeutic regimen^28^. Radiotherapy is currently applied intensively in the management of solid tumours, alone or in combination with other chemotherapy or immunotherapy treatments. As a new radiotherapeutic strategy, the FLASH effect induced by UHDR irradiation has been demonstrated in several in vivo animal models^29^. In order to produce a fundamental understanding of FLASH irradiation and beam interactions with cells, in vitro models need to be developed and investigated. In addition, the in vitro setup may be more reliable for certain experiments than animal testing since human cells can be used and the tumour microenvironment can be replicated under controlled conditions. The demonstration of the FLASH effect in vitro is challenging, but necessary in order to reveal a differential response between cancer and normal cells and identify the underlying molecular mechanisms involved. Phosphorylated γ-H2AX is the most sensitive marker of DNA damage and repair^30^. DNA methylation can rapidly reveal DNA double-strand breaks (DNA-DSBs), which are considered the most lethal form of DNA damage that compromises genomic stability^31^. Therefore, p-γ-H2AX foci analysis is the most robust method for investigating DNA damage and repair, while foci quantification is known as an accurate radiation biodosimeter^32^.

For the conventional X-rays used in radiotherapy, the molecular mechanisms involved in the generation and repair of DNA-DSBs are fairly well understood. Upon irradiation, the most damaging lesion produced is DNA-DSB. Genes involved in the cell cycle and growth control are upregulated^33^, and the progression of the cell cycle is stalled to allow the initiation of the DNA repair mechanism. One of the initial events upon DNA damage consists of the phosphorylation of thousands of γ-H2AX molecules flanking the damaged sites; these can be microscopically detected as foci. The radiation dose-dependent γ-H2AX phosphorylation reaction peaks 30 min after exposure and then declines over the following hours during the process of DNA repair^26^. The subsequent repair process is supported by the activation of key signalling molecules and pathways regulating post-irradiation cell fate. In addition to its role in activating antiapoptotic signalling, Akt stimulates DNA-DSB repair in tumour cells through the nonhomologous end-joining (NHEJ) repair pathway, thereby promoting tumour cell survival post-irradiation^34^. If DNA damage is persistent and intolerable by the cell, one of the death pathways will become activated^27^. The persistence of substantial numbers of γ-H2AX foci for 48 h after application of irradiation doses greater than 1 Gy allows the precise estimation of patient exposure when analysing peripheric blood lymphocytes or sampled skin^32^.

However, less is known on the irradiation dose ranges, fractionation regimens, and underlying mechanisms that can induce DNA damage and tumour cell death in regard to novel irradiation technologies. There are various pathways of regulated cell death (reviewed in ^35^), and many were found to be involved in the tumour cell response to radiation^36^, such as senescence^37^, apoptosis^38^, necroptosis^39^ and necrosis^40,41^. The mode of tumour cell death is crucial in vivo for determining whether anti-tumour immune priming occurs (known as the abscopal effect of radiotherapy)^42^. This systemic immune response triggered by local radiotherapy functions as an in situ cancer vaccination that contributes significantly to disease control.

Pioneering studies demonstrated that protonation accelerated by LPA resulted in the distinct formation of γ-H2AX foci at a proton dose of 20 Gy^43^ and dose-dependent biological damage induced in vitro in tumour cells^44^. On the other hand, the understanding of the DNA damage process in cells after electron irradiation delivered by LPA is still very limited^45,46^. Exposure to 100 fs single-shot 1 Gy of electron at a mean energy of 95 MeV demonstrated DNA damage in irradiated carcinoma cells according to the comet tail test. A very high dose rate demonstrated that a measurable assessment of the immediate and reversible DNA damage in cells could be performed at a single-cell level^16^. In another pioneering study, cancer cells exposed to electron bunches generated by LPA with an exponential spectrum of an average energy of 1.5 MeV at a cumulative dose of up to 2 Gy showed a radiobiological response by the induction of micronuclei and shortening of telomeres, as well as by the reduction of cell survival in blood samples and cancer tissue^47^.

Our studies represent the first in vitro experiments with cell co-cultures exposed to LPA electrons generated by the interaction of a high-intensity laser beam with gas jets. The cells were analyzed by immunofluorescence microscopy to quantify the DNA damage foci in response to the stress induced by radiological treatments using LPA electrons compared to pulsed X-ray irradiation. A non-uniform dose distribution on some discrete areas was revealed by Gafchromic films (GFs) in the case of LPA electron beam exposure. We detected double-strand DNA breaks in response to LPA electron radiation exposure. Using microscopic p-γ-H2AX foci counts, we showed that the number of foci induced 30 min after irradiation was consistent with the level of exposure, as revealed by dose mapping (Fig. 2); an increased frequency of nuclei with a high number of foci was detected in the sampled co-culture areas most affected by irradiation (Fig. 4; Fig. 5; Figure SI 4). In addition, in several investigated areas, more DNA-damaged cell nuclei were generated by LPA electrons than by X-ray irradiation at a cumulative dose one order of magnitude lower. Additionally, the nuclei exposed to the highest doses of the LPA electrons exhibited an increased number of DNA damage foci, as opposed to those exposed to pulsed X-rays. Next, we sought to investigate if a differential response of normal cells versus cancer cells occurred when exposed to the radiation source; if present, this differential response could account for the FLASH effect.

The benefit of radiotherapy is presently limited by the tolerance limit imposed by the normal organ tissues to increased doses of applied radiation. For example, radiation therapy was excluded from the list of treatment options for patients with intra-abdominal tumours (such as ovarian cancer-OC), due to high toxicity inflicted on the intestine, upon exposure to total abdominal irradiation^48^. Currently, there is a revived interest in the potential use of abdominal FLASH irradiation for the treatment of OC. Studies in mice have shown that it provides similar efficacy to conventional RT in controlling peritoneal metastases, while preserving intestinal function^49^. Moreover, the same team showed that abdominopelvic FLASH RT improves immunotherapeutic efficacy of anti-PD-1 checkpoint inhibition in preclinical models of OC^50^. A recent study evaluated the impact of UHDR FLASH total body irradiation (TBI) on blood cancers and normal haematopoiesis in a humanized mouse model^51^. The authors investigated T-cell acute lymphoblastic leukaemia (T-ALL) progression and normal human haematopoiesis, upon FLASH-TBI versus CONV-TBI; FLASH (4 Gy) was produced using a prototype 6 MeV electron beam linear accelerator. Leukaemic cells extracted from irradiated mice were either cultured in vitro or transplanted into secondary recipients. The number of proliferating cancer cells was 4 times lower for FLASH-RT than for CONV-RT-exposed cells 7 days after culture; the leukaemic tumour burden was significantly decreased in secondary recipients, with 6% of leukaemic cells (CD45^+^CD7^+^) in the bone marrow (BM) of mice bearing FLASH-RT cells and > 90% of leukaemic cells in mice receiving CONV-RT-exposed cells. Haematopoiesis and normal haematopoietic stem/progenitor cell functions, were partially spared upon FLASH-RT irradiation, while CONV-RT completely abolished these functions. Hence, the detrimental effects of CONV-RT on normal cells were reduced by using FLASH-RT setup, while increasing the benefit of radiation treatment^51^. Interestingly, when secondary transplantation was performed from a mixture of leukaemic and normal human haematopoietic cells, FLASH-RT was superior in preventing cancer relapse but impeded normal haematopoiesis from occurring, potentially due to alteration of the BM niche in the humanized mouse model^51^. Normal tissue sparing by FLASH-RT in the UHDR regimen has been previously demonstrated in another acute responding organ (gastrointestinal tract), as well as in late responding organs (lung, brain, skin), relatively independent of the ionizing radiation source type^52,53^.

In our present study, we concomitantly exposed melanoma cancer cells and normal melanocytes as in vitro co-cultures to FLASH irradiation via VHEE generated by the interaction of a high-intensity PW laser beam with a supersonic gas jet. The differential responses of cancer and normal cells were detected in discrete areas of the exposed specimen. Notably, we identified a discrete sample area where the VHEE exposure conditions had less DNA damage-inducing impact on the non-cancerous NHEM cells than on the A375 melanoma cells (Fig. 5 – top area of the PW2-irradiated slide). This result may be an essential step towards the possibility of understanding the fundamental mechanisms of the FLASH effect for our PW laser electron beam generation system. However, further VHEE setup optimization is needed to enhance the system stability and enable experimental reproducibility to determine an accurate correlation between the experimental parameters and cell responses. This phase is key for validating the potential of the VHEE generated by LPA and can be further developed and potentially tested in clinical trials.

Recent results obtained with VHEE produced by an LPA accelerator in water phantoms have shown that a significant dose relevant for preclinical studies can be delivered to deep-seated targets^23^. The cumulative dose delivered to the target was up to 1.6 Gy and was achieved by applying a few hundred shots. Thus, deep-seated tumours could be targeted by VHEE beams, as the dose deposited in the tissue can be higher than doses currently delivered by conventional radiotherapies^46^. Furthermore, the dose distributions generated by VHEE electrons are less affected by body inhomogeneities than those generated by protons^54^. Although FLASH-RT technology is still in its infancy from the viewpoint of medical application, the first case studies on human patients have been reported^55,56^, and three phase 1 clinical trials are ongoing to test primarily toxicity in patients with thoracic bone metastases or melanoma (reviewed in ^57^). However, the knowledge gap remains important, and safe and efficient parameters need to be determined for translation into the clinic and for the generation of a positive response. To address this issue, extensive research is needed to understand the dose-dependent mechanisms of action of electron FLASH-RT on cells^58^ and to determine which are the prerequisite patient characteristics that classify patients as responders. The studies of Chabi S and collaborators^51^ presented above revealed intrinsic genetic cues that differentiated radiation-sensitive from radiation-resistant leukaemic cancer cells.

In perspective, ultrafast radiation biology will likely be developed as a new domain in an emerging interdisciplinary field, along with the development of new ultrashort radiation sources and advanced cancer therapeutic strategies. Moreover, very high dose rates and ultrashort dose fractionations may be proposed in combination with advanced chemotherapeutic strategies because they enable real-time control of the amplified radio-sensitivity^59^.

Our results represent the first demonstration of a differential response of tumour and normal cells exposed the FLASH irradiation using an in vitro model. The experiments confirm the DNA damage-producing capacity of the laser-driven high-energy–dose electrons produced by a table top high power laser – plasma accelerator in cancer cells for future FLASH-RT applications. These findings may contribute to the development of either new cell culture approaches to explore fundamental understanding of cell induced FLASH effect or to the employment of new strategies for the improvement of stability and uniformity of new FLASH irradiation sources.

## Materials and methods

### Cell culture

Radiation-resistant A375 human metastatic melanoma cells and normal human epidermal melanocytes (NHEMs) were obtained from ATCC (#CRL-1619) and Lonza (#CC-2504), respectively. The A375 cells were grown in high-glucose (HG) Dulbecco’s modified Eagle’s medium (DMEM) supplemented with 10% heat-inactivated foetal bovine serum, 1% L-Glutamax, and 1% penicillin/streptomycin; all reagents were purchased from Thermo. The NHEM cells were grown in MBM-4 medium (#CC-3250) supplemented with SingleQuots (#CC-4435) and manipulated according to the manufacturer’s instructions. The cells were maintained in a humidified incubator with 5% CO_2_ at 37 °C. One day before the irradiation experiments, the cells were plated in 9 cm^2^ polystyrene SlideFlasks (#170920, Thermo) in a 1:2 ratio of NHEM:A375 cells (420,000:840,000 cells) and the corresponding 1:1 media mix; the cells reached confluency within 24 h. Immediately before irradiation, the SlideFlasks were filled with 19 mL of prewarmed complete DMEM HG media. For cell exposure, we used SlideFlask containers, as they provide a high number of cells on large areas for exposure to VHEE or X-ray irradiation and the possibility of removing the flask chamber from the slide supporting the cells to be analysed with high resolution by fluorescence microscopy. For PW room environmental exposure condition testing, the cells were seeded directly onto the plastic surface of 24-well plates (Corning) and placed in the irradiation room outside the beam area during the experiment (PW env), and another well plate was left in the incubator (untreated). The technical control samples were seeded on 12 mm coverslips (Marienfeld) plates and placed in 24-well plates (Corning); the untreated and 2 μM cisplatin (CisPt)-treated cells were compared to set the thresholds for the analysis of the DNA damage-induced foci.

### VHEE setup

**Figure 6:**
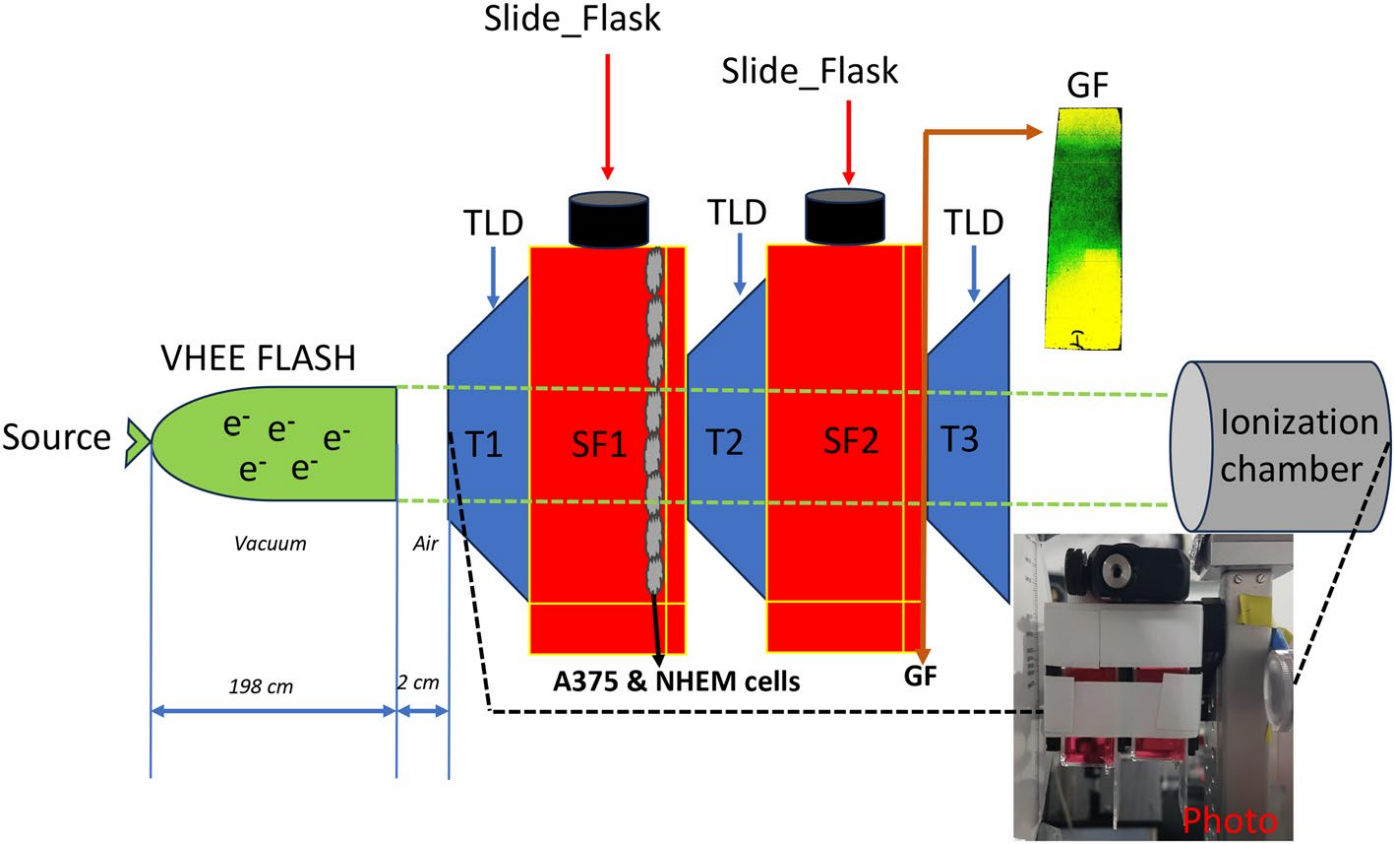
Laser-gas target experimental setup for the generation of the accelerated electron beams and irradiation of the cell co-cultures in vitro.

The high-intensity laser beam was focused with an off-axis parabolic mirror with a 3.2 m focal length (f# 27) on a supersonic gas jet consisting of 99% He + 1% N_2_^60^. The quasi-monoenergetic electron energy distributions were measured with a spectrometer equipped with a Pb collimator and magnetic dipole of 0.8 T, which was set up into the electron beam at the extension of the interaction chamber. Figure 6 shows a sketch and an image of the experimental setup used for LPA VHEE cell irradiation (LPA experimental setup and photo in Figure SI 1). Three irradiation sessions were performed. Two experimental configurations were chosen to allow simultaneous irradiation of the two containers with cells grown either in the first or both containers; Fig. 6 shows the configuration in which cells were placed only in the first container. In our set-up, three thermoluminescent dosimeters (TLDs) were introduced: one was placed in front of the first container, the second was placed between the two containers, and the third was placed after the second container (see TLDs 1, 2, and 3 in Fig. 6). Before reaching the first TLD, the electron beam travelled approximately 198 cm in vacuum, and then it travelled successively through a 60 µm Al foil, a 1 cm thick glass window, another 60 µm Al foil, 0.5 mm cardboard, the scintillator screen LANEX and 2 cm in air.

### Dosimetry measurements

**Figure 7:**
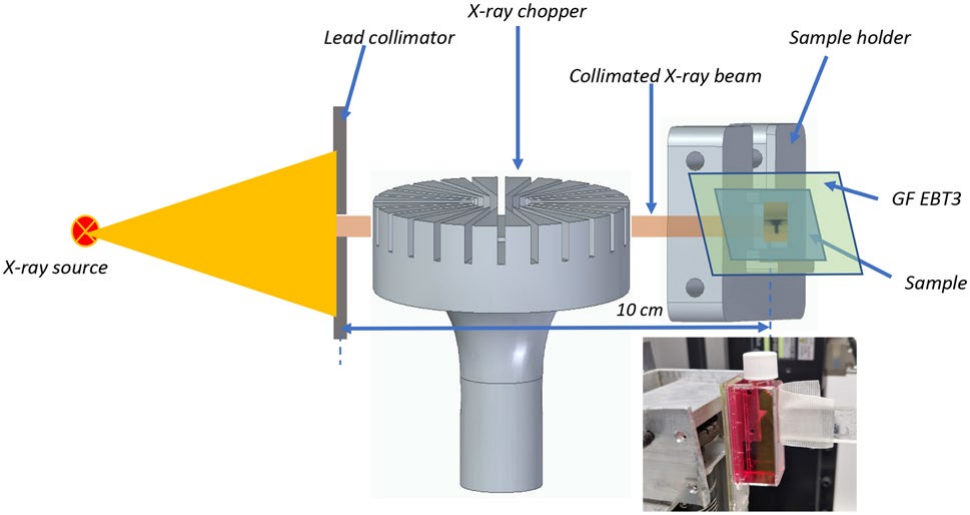
Schematic representation of the pulsed X-ray irradiation setup.

Electron energy distribution measurements were carried out using a spectrometer equipped with a collimator and magnetic dipole, and the sample was placed into the electron beam at the extension of the interaction chamber. To estimate the dose, we used three independent dosimetry systems: (i) a dedicated ionization chamber for electron beam dosimetry (Advance Markus type) to monitor the total applied dose for each experiment; (ii) three TLDs (Panasonic type) to monitor the dose through the cell containers; and (iii) a Gafchromic film (GF-EBT3 type) to provide correlation with the dose distribution on the microscopic slide (Fig. 7). All dosimetry systems were calibrated before the experiment. The TLD detectors were prepared and read by an accredited third-party laboratory.

An Advance Markus ionization chamber type TN34045, which is an air-vented plane-parallel ionization chamber, was used to measure the total dose absorbed by water in electron beams. Compared with other chambers, the Advance Markus chamber demonstrated the best performance for the FLASH doses^7^. The chamber was connected to a UNIDOS dosimeter from PTW. Measurements were performed with a continuous charge collection low-dose regime.

The TLD Panasonic thermoluminescent dosimeters contained 4 thermoluminescent elements (two of *Li*_2_*B*_4_*O*_7_:*Cu* (with *Z*_*efectiv*_ close to human tissue) and two of *CaSO*_4_: *Tm* (high sensitivity)) to determine the average radiation energy and the dose recorded by the dosimeter, using all the information provided by the 4 elements. A digital thermo-luminescent dosimeter (TL Badge) (model Panasonic UD-716) was used as the reader. The sample stack was perpendicular to the electron beam axis. The relative depth doses were measured. No beam collimator was used.

The Gafchromic film was a self-developed EBT-3 designed for the measurement of absorbed doses of ionizing radiation. These films are widely used in medical radiotherapy departments for quality assurance and dose measurements. The film darkening was measured using an integrated densitometer in a dedicated EPSON Expression 11000XL professional scanner with a resolution of 4800 dpi in transmission mode^61^.

### Pulsed X-ray setup

The setup used for delivering pulsed X-rays consisted of an X-ray chopper made of steel with a brass infusion, which had 12 slits (each with a height of 8 mm and a width of 1.5 mm) placed at 15-degree intervals from each other. It also included a sample holder that positioned the sample at a distance of 10 cm from the head of the X-ray source. A pair of lead collimators, each 2 mm thick, was placed in front of the X-ray head to reduce the size of the X-ray beam and X-ray scattering. The chopper was rotated by a high-speed brushless DC motor connected to an electronic speed controller (ESC) unit. The rotational speed was controlled with the help of an electronic board that provided a pulse width modulated (PWM) signal. During the irradiation experiments, the servo position was set with a pulse width of 1469 µs, which corresponded to an X-ray pulse frequency of 1.4 kHz. The X-ray source was set to a voltage of 200 kV and a current of 100 µA, and a copper foil with a thickness of 0.2 mm was placed in front for radiation filtration. For these X-ray source operating parameters, under continuous exposure, a dose rate of 26 mGy/s at a distance of 10 cm was measured by an ionization chamber connected to a standard UNIDOS dosimeter. In the case of pulsed X-ray irradiation conditions, the doses delivered to the samples were estimated using Gafchromic EBT3 films that were placed at the back of the cell culture Slide Flask (Fig. 7). The samples were irradiated with pulsed X-rays for 15 min and 31 s, and according to Gafchromic EBT3 measurements, the irradiated flask region (symbolled with a T sign in Fig. 7) received a total dose of 2.4 Gy. The EBT3 films were previously irradiated with precisely delivered doses, and calibration curves were determined from the radiochromic film darkening and were proportional to the absorbed dose. The setup for pulsed X-ray irradiation was kept identical between irradiation campaigns.

### Quantification of the biological effects

After irradiation (at the PW laser or X-ray facilities) at room temperature (RT), the cells were allowed to recover in the incubator for 30 min and then washed once with phosphate-buffered saline (PBS) before being fixed together with the controls using 4% p-formaldehyde (PFA) for 30 min at RT. The cells were washed twice with PBS and stored at 4 °C until further processing.

To evaluate the double-strand DNA breaks in response to radiation exposure, we examined the appearance of the nuclear p-γ-H2AX foci using specific mouse monoclonal antibodies for the Ser140 phosphorylated form of γ-H2AX (#MA1-2022, Invitrogen, Thermo). The fixed cells were first permeabilized using 0.02% Triton-X-100 in PBS for 3 min, followed by 3 washes in PBS. Next, 0.5% BSA-PBS was added for a 1 h incubation to block nonspecific binding sites. The antibody was used at a 1:200 dilution in the blocking buffer for 30 min at RT, followed by a series of PBS washes and incubation with AlexaFluor 594-conjugated donkey anti-mouse secondary antibodies (#A21203, Invitrogen, Thermo). Finally, the nuclei were labelled with Hoechst 33342 (#H21491, Molecular Probes, Thermo) for 1 min, and after thorough washing, the samples were mounted using FluorSave Reagent (#345789, Millipore) and left to dry overnight. The slides were scanned using the TissueFAXSiPlus automated imaging system (TissueGnostics, Vienna, Austria), and the manual focus option was selected to generate the optimal fluorescence images given the planarity imperfections of the SlideFlask polystyrene surface. Stitched overview images were generated for each slide during previewing at 5 × magnification. The regions of interest (ROIs) were created using the rectangle tool for each area to be investigated, considering irradiation areas of interest; similar ROIs were delineated on controls. Next, ROIs were obtained using a 63 × oil objective, excluding the environmental controls; the environmental controls were examined using 20 × air due to the objective working distance limitations for focusing into the 24-well plate bottom.

### Image cytometry analysis

**Figure 8:**
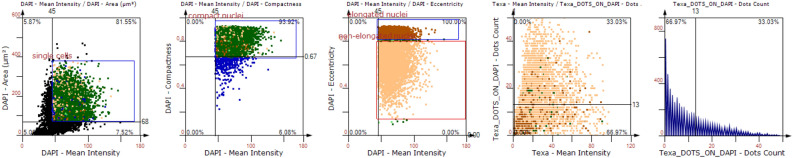
Gating strategy used for image cytometry analysis in TissueQuest 4.0.1.0140 for cells exposed to different irradiation conditions. Scattergrams of cisplatin (CisPt)-treated positive control co-cultures are given here as examples. CisPt is a known inducer of DNA damage and is used to treat various types of cancers.

To determine the percentage of p-γ-H2AX^+^ cells, the mean fluorescence intensity (MFI) of the p-γ-H2AX signal, and the number of p-γ-H2AX foci per nucleus, the samples were scanned with the TissueFAXS Slides 3.5.5.0129 module using a 63 × oil objective. The signals recorded through the DAPI (λ_ex_ 360 nm/λ_em_ 462 nm) and TxRed (λ_ex_ 568 nm/ λ_em_ 603 nm) filters were further analysed upon nuclear segmentation using TissueQuest software version n. 4.0.1.0140 (TissueGnostics, Vienna, Austria, URL: https://tissuegnostics.com/products/single-cell-analysis/tissuequest,). Using the gating strategy exemplified in Fig. 8 for the CisPt positive control^62^, DAPI-area, and DAPI-mean intensity parameters were used for single cell recognition, followed by DAPI-compactness *vs.* DAPI-mean intensity to select compact nuclei and exclude irregularly shaped artefacts. Next, the gated compact nuclei were visualized in a DAPI eccentricity *vs.* DAPI-mean intensity scattergram to identify elongated nuclei (high eccentricity) of the NHEM cells and non-elongated nuclei (low eccentricity) of the A375 cells. The Texa-Mean Intensity and Texa_DOTS_ON_DAPI parameters were used to quantify the p-γ-H2AX MFI (Figure SI 4fig) and the number of foci (Fig. 4), respectively.

The thresholds for every parameter were optimized using backward gating to examine the images of the segmented cells within each population. Additionally, the setup was validated using negative control samples (untreated, non-irradiated). Statistical reports and dot statistics were generated to extract the quantification values for each sample under investigation. A total number of 7370–15,739 cells were contained in each condition, depending on each region of interest (ROI) cellular content. Upon removal of artefacts by drawing exclusion regions and filtering of data through sequential gating (Fig. 8, first 3 scattergrams), the number of nuclei left for p-γ-H2AX quantification were between 4473 and 10,629 (see Table SI 1).

### Statistical data analysis

Experimental data are reported of two serial replicates (PW1 and PW2) from one irradiation session out of two performed. The image cytometry data were imported into GraphPad Prism 9.5.1 (528) to generate bar graphs of the obtained values and to analyse the statistical differences between the PW or X-ray irradiation conditions, and the control non-irradiated sample. To evaluate potential differences in double-strand DNA breaks induction, statistical comparisons were performed at 30 min post-irradiation by Mann–Whitney test. A *p* value of < 0.05 was considered to indicate statistical significance. All adjusted *p* values are represented on the graphs as star symbols and explained in figure legends.

### Supplementary Information

Supplementary Information.

Supplementary Video 1.

## Data Availability

All data are available within the Article and Supplementary Files or available from the corresponding authors upon reasonable request.
