# Supplementary material for: First in vitro cell co-culture experiments using laser-induced high-energy electron FLASH irradiation for the development of anti-cancer therapeutic strategies
Source: Sci Rep. 2024 Jun 27;14:14866. doi: 10.1038/s41598-024-65137-7 (PMC11211417; doi:10.1038/s41598-024-65137-7)
Supplement: Supplementary file 1 — Supplementary Information. [file 41598_2024_65137_MOESM1_ESM.docx]

SUPPLEMENTARY INFORMATION

*First in vitro cell coculture experiments using laser-induced high-energy electron FLASH irradiation for the development of anti-cancer therapeutic strategies*

*LPA experimental setup*

The high-intensity laser beam was focused with an off-axis parabolic mirror of a 3.2 m focal length (f# 27) on a supersonic gas jet consisting of 99% He + 1% N2. Electron energy distributions have been measured with a spectrometer equipped with a Pb collimator and magnetic dipole of 0.8T, set up into the electron beam at the extension of the interaction chamber. Fig. SI 1 presents a detailed LPA experimental setup and a photo of cell culture containers placed in between three TLDs. In our set-up, three thermoluminescent dosimeters (TLD) were introduced: one was placed in front of the first container, the second TLD between the two containers the third TLD was placed after the second container (see TLD 1, 2, 3 in Fig. 4). Before reaching the first TLD, the electron beam travels approximately 198 cm in a vacuum, after which it travels successively through a 60 µm Al foil, a 1 cm thick glass window, another 60 µm Al foil, 0.5 mm cardboard, the scintillator screen LANEX and 2 cm in air.


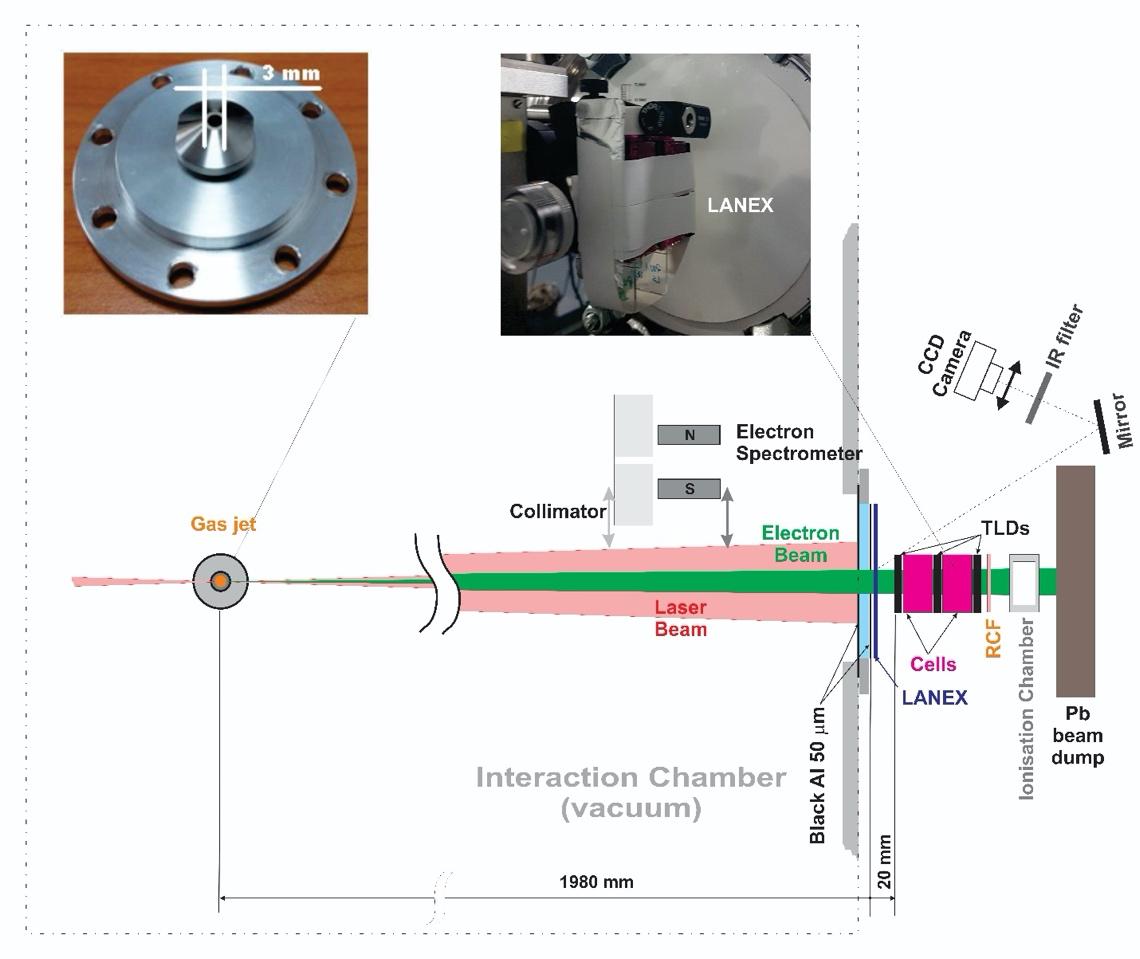


*Figure SI 1: Schematic diagram of the laser-gas target experimental setup for the generation of accelerated electron beams and the irradiation of cell co-cultures in vitro*

***Dosimetry measurements:***

For relative measurements of the doses delivered to the cells, we used three different dosimetry systems. The dose values provided are as follows:

i) Advanced Markus ionization chamber: I. 149.9 mGy and II. 154.8 mGy

ii) TLD placed in front, middle, and behind cell containers: I. 1107.28 mGy, 627,48 mGy and 400,97 mGy; II: 1263,08 mGy, 748,28 mGy and 429,65 mGy

iii) Gafchromic films placed behind the stack: I. 195 mGy and II. 170 mGy.

i) The Advance Markus ionization chamber type TN34045, an air-vented plane-parallel ionization chamber was used to measure the total dose absorbed to water in electron beams. It is generally used for dose measurements in electron beams with energies between 2 and 45 MeV. For other energies, proper corrections have to be applied. The measurement volume is 0.02 cm3. The chamber's nominal working voltage is between 50 and 300 V. In our experiment, 300 V was used for dose measurements. The entrance chamber wall material is polyethylene and has a thickness of 0.03 mm. The electrode material is PMMA coated with graphite with a diameter of 5 mm. The environmental conditions for using the chamber are temperature between 10 and 40°C, humidity between 10% and 80%, and air pressure between 700 and 1060 hPa. The chamber was calibrated to the primary Co-60 beam at PTB. The presented results are not corrected to dose per pulse and beam energy effects.

ii) TLD Panasonic thermoluminescent dosimeters contain 4 thermoluminescent elements, two of 𝐿𝑖2𝐵4𝑂7: 𝐶𝑢 and two of 𝐶𝑎𝑆𝑂4: 𝑇𝑚 to determine the average radiation energy and the dose recorded by the dosimeter, using all the information provided by the 4 elements. The 𝐿𝑖2𝐵4𝑂7: 𝐶𝑢 chips are sensitive to β, γ, n beam in the 100 µ𝑆v −10 𝑆v dose range. The 𝐶𝑎𝑆𝑂4: 𝑇m chips are sensitive to β, γ, and γ beams in the dose range of 10 µ𝑆𝑣 −500 𝑚𝑆v and 10 µ𝑆𝑣 −50 𝑚𝑆v respectively. A beam energy range from 10 keV to 10 MeV is provided by the manufacturer. The Panasonic UD-716 reader was a multi-purpose digital thermo-luminescent dosimeter (TL Badge). The heating method employed was PANASONIC original infrared heating. The built-in microcomputer enables full corrections and arithmetic handling of the measured data and performs data transmission to and from the external computer. Thus, the reader offers high quality, high performance, and the full range of functions.

iii) The gafchromic film was EBT-3, designed for the measurement of absorbed doses of ionizing radiation. The dynamic range of this film is designed for best performance in the dose range from 0.1 to 10 Gy. These films are widely used in medical radiotherapy departments for quality assurance and dose measurements. These films have a near-tissue equivalent. They are not sensitive to radiation incident energy. The calibration curve in X-ray and high energy electron beam shows a similar value. The EBT-3 gafchromic films are self-developing. The film darkening was measured using an integrated densitometer in a dedicated EPSON Expression 11000XL professional scanner with a resolution of 4800 dpi in transmission mode.

The higher dose measured with TLD as compared to the dose measured by the ionization chamber may suggest the presence of considerable electron numberscwith energies below 12 MeV which were stopped in the sample stack.The presence of the high energy electrons in the spectrum is eventually revealed with the gafchromic and ionization chamber dose measurements that pass through the entire sample stack. These electrons have been measured with gafchromic film and ionization chamber behind the entire sample stack and show similar values corresponding to measurements uncertainty of both independent dosimetry systems.

**Monte Carlo simulation - geometry and details:**

Monte Carlo simulation stands as the sole viable method for accurately determining dose distributions in samples exposed to Very High Energy Electron (VHEE) beams. This is due to the unavailability of a direct measurement approach for verifying VHEE electron beams, given that such beams are not currently very accessible for experimental validation.

The data on depth dose distribution of sample stack irradiated by VHEE have been computed by Monte Carlo code PENELOPE2014, which is a Fortran-77 subroutine package able to simulate electron, positron, and photon transport through matter.

Energetically calibrating the electron beam employed in irradiation experiments is challenging to achieve through experimental measurements. Considering that the absorbed dose depends on the energy of the irradiation beam, Monte Carlo simulations were conducted in this case to better comprehend the doses measured by TLD dosimeters during the irradiation experiments of the sample stack.

The geometry of the irradiated sample in the simulation program was defined as simply as possible to closely emulate the sample stack used in experiments while ensuring a reasonably short simulation time. Thus, the cell flask phantoms were defined as right rectangular prisms made of water with a thickness of 2 cm (green and orange body) and surrounded by a layer of polyethylene (red and blue body) with a thickness of 0.02 cm. TLD (thermoluminescent dosimeter) materials with a thickness of 0.1 cm were specified at both ends of the two phantoms and between them. The TLD dosimeter was defined as a homogeneous medium of 𝐶𝑎𝑆𝑂4: 𝑇𝑚 with 1% at. concentration of Tm. The simulation starts with a monoenergetic electron beam with energy of 10 or 100 MeV hitting perpendicular to the surface of the sample stack which is placed at a distance of 1,8 m from the beam source (as in experimental condition). The cut-off parameters, i.e. the energy cut-off for electron, photon, and positron trajectory tracing through each simulated material (EABS1:3) were set at 10 keV while the cut-off parameters for the elastic scattering were set as C1= C2 =0.05. For the bremsstrahlung emission and inelastic collisions, the cot-off was also set at 10 keV (WCR =WCC =10 Kev). No variance reduction or interaction forcing techniques were used. A total of 1x108 particles were simulated for each run time.

Fig. SI 2 presents the absorbed dose distributions through the entire sample stack. For both electron energies, the absorbed dose through TLD dosimeters is higher than in the water phantoms and can be observed as three valleys centered at the hitting surface (0 cm), in the middle of the sample stack (2.2 cm), and at the end of the sample stack (4.5 cm). This is because the density and the effective atomic number of TLD material are higher than water phantom and polyethylene. For comparison reasons, the dose measured by TLD dosimeters during irradiation experiments is also presented in the graph of Fig. SI 2.

From Monte Carlo simulations, we observe that the energy deposition of 10 MeV electrons gradually drops within the sample stack compared to 100 MeV electrons which show a quasi-uniform distribution over the whole stack depth. These results are supported by studies previously reported in the literature1, which indicate that with an increase in the irradiation beam energy, the maximum absorbed dose occurs at greater depths. For a geometry similar to our cells stack, the highest energy absorption occurs with 10 MeV electrons and reaches a maximum at a depth of around 4 cm. Based on the dose depth distribution simulated in Fig. SI 2, one may suppose that the electron pulse has a wide energy distribution with a significant component up to 10 - 12 MeV that might explain the decreasing tendency of doses measurement by TLD dosimeters and a higher energy part (over 50 MeV) that was captured by the ionization chamber and the Gafchromic films.


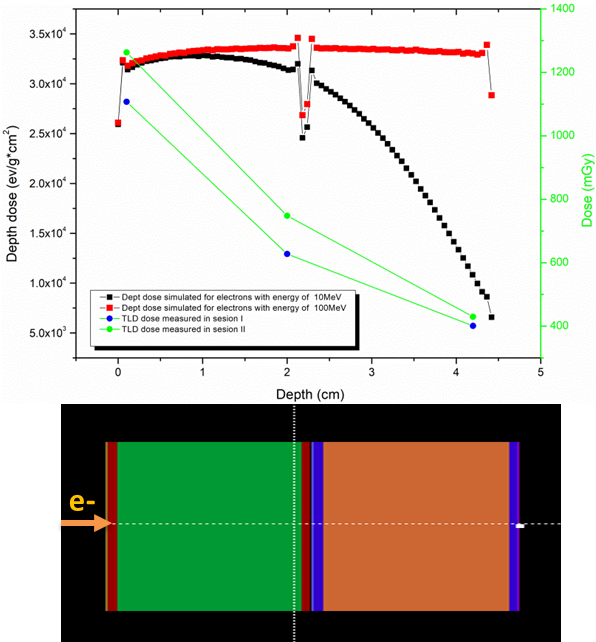


*Figure SI 2:Depth dose distribution through sample stack (top image) and the corresponding simulated geometry for stack sample(bottom image).*

***Image cytometry data processing of melanoma and normal melanocyte*** ***co-cultures irradiated by PW-induced***  ***electrons or by pulsed X-ray***


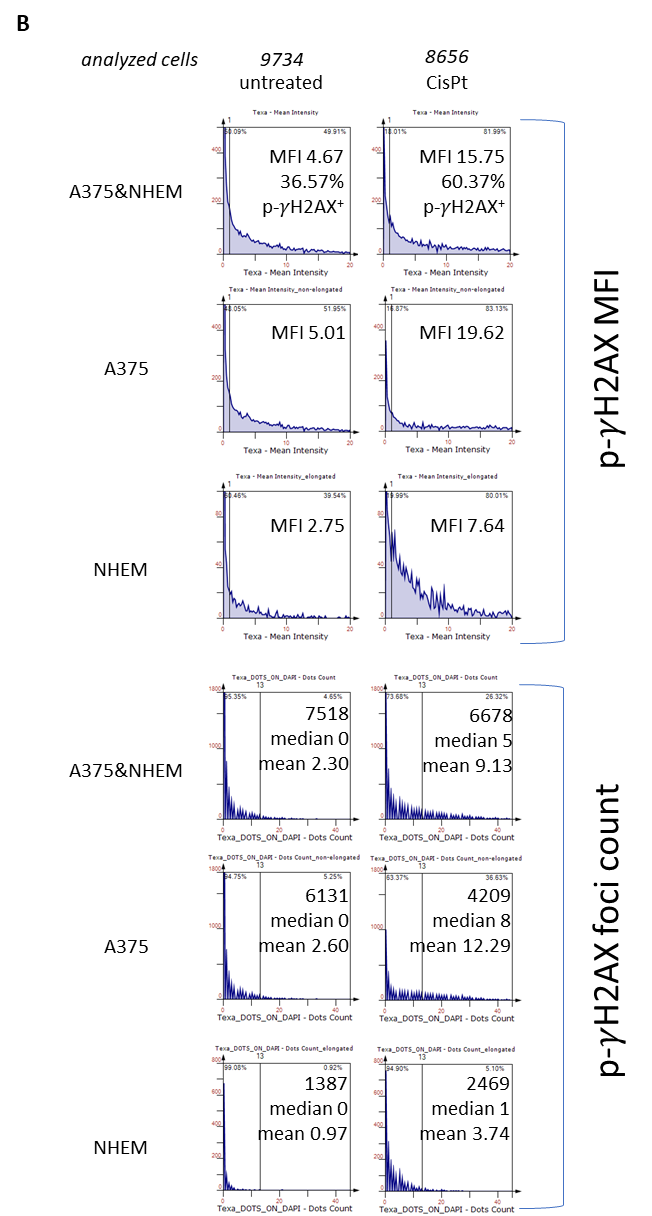

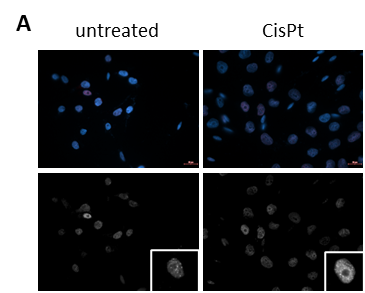

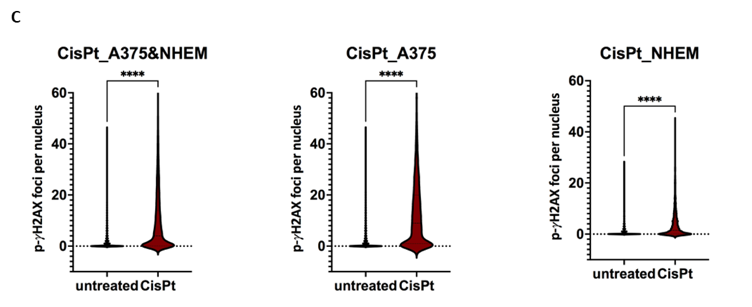


*Figure SI 3 (related to Figure 8): Analysis of A375 melanoma and NHEM co-cultures upon CisPt treatment versus untreated control. (A) DNA damage response revealed as p-γ-H2AX foci at 24h post-exposure to CisPt versus not exposed (untreated) negative control. Immunofluorescence microscopy images of overlapped DAPI and TxRed signals (top), as well as grayscale images of TxRed signal (bottom) of representative field of views (FOVs) in each condition are depicted. The p-γ-H2AX foci are visible in detailed insets (bottom right). Scale bar = 20m; (B) Image cytometry histograms depicting distribution of Texa – Mean Intensity values in the investigated cell populations (co-culture vs. melanoma in co-culture vs. melanocytes in co-culture) and quantification of the percentage of p-γ-H2AX+ cells, median fluorescence intensity (MFI) of p-γ-H2AX signal (top panel of grouped histograms), as well as distribution of p-γ-H2AX foci count with the associated median and mean values for each condition tested (bottom panel of grouped histograms). (C) Violin plots of „number of foci per nucleus” values frequency distribution for CisPt treated cells vs. untreated control. Graphs depict comparisons between treatments for overall A375&NHEM co-cultures, for A375 cells in the co-culture, and for NHEM cells in the co-culture, respectively. Statistical differences were determined using unpaired two-tailed Student’s t-test* *(n = 8656-9734 nuclei, **** p<0.0001).*

*
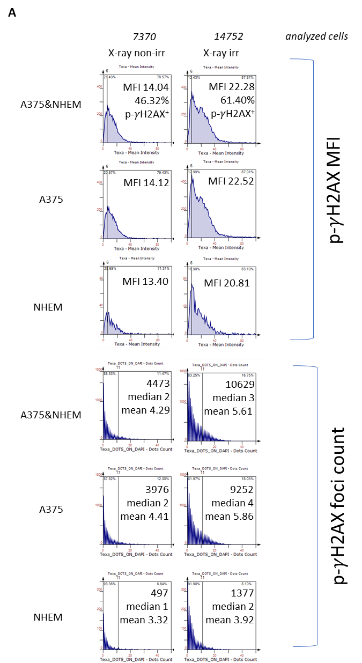
*

*
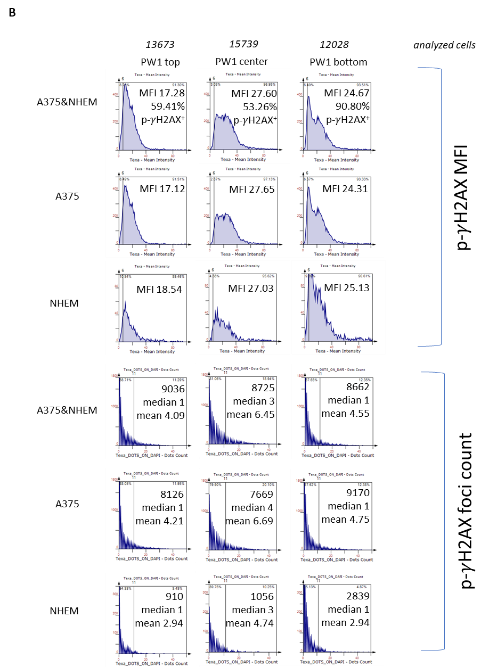
*

*
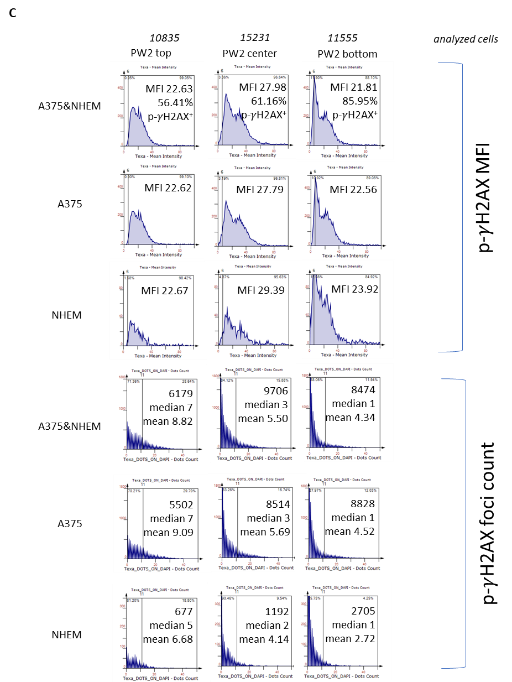
*

*Figure SI 4 (related to Figure 4): Analysis of A375 melanoma and NHEM co-cultures upon PW irradiation vs. pulsed X-ray or untreated controls. (A-C) Image cytometry histograms depicting distribution of Texa – Mean Intensity values in the investigated cell populations (co-culture vs. melanoma in co-culture vs. melanocytes in co-culture), median fluorescence intensity (MFI) of p-γ-H2AX signal and percentage of cells expressing nuclear p-γ-H2AX, for each condition tested: (A) pulsed X-ray vs. non-irradiated cells, (B) PW1 irradiated cells – top vs. center vs. bottom ROIs, (C) PW2 irradiated cells – top vs. center vs. bottom ROIs.*

*Table SI 1 (related to Materials and Methods): Number of analyzed cells for each irradiation condition*

| **Area in the sample/Sample** | **Number of total cells analyzed (*n*)** | **Number of A375 cells analyzed (*n*)** | **Number of NHEM cells analyzed (*n*)** |
| --- | --- | --- | --- |
| non-irradiated | 4473 | 3976 | 497 |
| irradiated/X-ray | 10629 | 9252 | 1377 |
| top area/PW1 | 9036 | 8126 | 910 |
| center area/PW1 | 8725 | 7669 | 1056 |
| bottom area/PW1 | 8316 | 7395 | 921 |
| top area/PW2 | 6179 | 5502 | 677 |
| center area/PW2 | 9706 | 8514 | 1192 |
| bottom area/PW2 | 8261 | 7451 | 810 |

**References**

1. Bliznyuk, U. *et al.* Electron Beam Processing of Biological Objects and Materials. in (ed. Artun, O.) Ch. 5 (IntechOpen, 2023). doi:10.5772/intechopen.112699.
